# A Modified 90-Degree Distal Chevron Metatarsal Osteotomy for Correcting Moderate Hallux Valgus Deformity

**DOI:** 10.3390/jcm12216902

**Published:** 2023-11-02

**Authors:** Min Gyu Kyung, Gil Young Park, Hanbual Yang, Dong Yeon Lee

**Affiliations:** 1 Department of Orthopedic Surgery, Seoul National University Hospital, Seoul 03080, Republic of Korea; mgkyung@snu.ac.kr (M.G.K.); hanstartnow@gmail.com (H.Y.); 2 Department of Orthopedic Surgery, SNU Seoul Hospital, Seoul 07803, Republic of Korea; gilyoungpark@gmail.com; 3 Department of Orthopedic Surgery, Seoul National University College of Medicine, Seoul 03080, Republic of Korea

**Keywords:** foot, metatarsal bones, hallux valgus, distal chevron metatarsal osteotomy

## Abstract

Various modifications of distal chevron metatarsal osteotomy (DCMO) have been introduced for correcting moderate hallux valgus deformity; however, the amount of correction may be limited, and complications, such as the recurrence of the deformity and avascular necrosis of the metatarsal head, have been a problem. This study aimed to present a modified 90-degree DCMO technique that overcomes the previously reported shortcomings and to report a successful short-term radiographic outcome. Sixty-eight consecutive patients who underwent the operation with our modified DCMO technique and twenty-two consecutive patients with the conventional DCMO technique (control group) were retrospectively analyzed. The radiographic measurements were evaluated preoperatively, at two months post operation, and at the final follow-up. Both groups showed a significant correction of the hallux valgus angle, first–second intermetatarsal angle, distal metatarsal articular angle, and sesamoid position at the final follow-up, while the amount of correction was significantly greater in the modified DCMO group. In both groups, there were no cases of complications such as avascular necrosis of the metatarsal head, nonunion, and surgical site infection appearing throughout the follow-up period. Therefore, the modified 90-degree DCMO technique is effective and safe, which could serve as a favorable option to treat moderate hallux valgus deformity.

## 1. Introduction

Hallux valgus deformity is characterized as a complex three-dimensional deformity existing in the area of articulation between the first proximal phalanx and distal metatarsal head [1]. The development of hallux valgus deformity can sometimes be linked to genetic factors, with some patients having a familial tendency of the condition [2]. However, frequently wearing high-heeled shoes or shoes with a narrow and pointed front can also contribute significantly to its onset. Patients often complain of pain in the bunion, which protrudes on the medial side, and experience difficulty in wearing shoes due to it. Initially, a conservative treatment is implemented. Using orthosis like a silicon toe spacer or wearing shoes with a wide toe box can alleviate some of the pain. However, there is no evidence that the progression of hallux valgus can be prevented or reverted to its original state. Therefore, surgical intervention becomes necessary to ultimately address hallux valgus that does not respond to conservative treatments.

The surgical approach varies depending on the severity of the hallux valgus. The severity is traditionally classified into mild, moderate, and severe based on radiographic measurements such as the hallux valgus angle (HVA) and first–second intermetatarsal angle (IMA). In mild-to-moderate cases, distal chevron metatarsal osteotomy (DCMO) has gained popularity [3] and demonstrated satisfactory results with the advantages of technical simplicity and a short rehabilitation period [4,5].

However, it is believed that the amount of correction is limited compared to that of proximal metatarsal osteotomy [6], and if the capital fragment is displaced in a parallel fashion, theoretically, the distal metatarsal articular angle (DMAA) and medial sesamoid position will not change much after the operation [7,8]. Coughlin and Matsumoto et al. pointed out that the presence of an increased DMAA was closely related to the undercorrection of the deformity [7,9]. In addition, the failure of correcting the subluxated sesamoid is considered an important factor of the recurrence of hallux valgus [10]. Moreover, possible complications such as avascular necrosis (AVN) of the metatarsal head have been reported [11,12].

In order to overcome these possible concerns, here, we present a modified 90-degree DCMO technique for correcting moderate hallux valgus deformity. A previous technical note introduced a reversed L-shaped DCMO, where the long plantar arm was cut perpendicular to the short dorsal arm [13]. In another study by Donnelly et al., they reported a different configuration of DCMO with short dorsal and long plantar arms [14]. While these studies reported a similar cutting method to ours, no direct comparison of radiographic outcomes between other techniques was available. We aimed to provide detailed figures of our modified technique and report a successful short-term radiographic outcome. Furthermore, we attempted to compare the radiographic outcome with that of conventional DCMO as a control group to determine whether there were any differences in the amount of correction.

## 2. Materials and Methods

This study was a retrospective level III case–control study and was approved by Seoul National University Hospital Institutional Review Board (IRB number: H-2111-034-1269). Owing to the retrospective nature of the study, the requirement for informed consent was waived by Seoul National University Hospital IRB. All research protocols were carried out in accordance with the Declaration of Helsinki.

### 2.1. Study Subjects

A total of 280 consecutive patients (340 feet) with hallux valgus deformities who underwent correction surgery in our institute between 2009 and 2021 were retrospectively reviewed digitally via the hospital information system. The inclusion criteria were as follows: HVA > 15 degrees, persistent forefoot pain even after at least six months of conservative treatment, and age over 20. The exclusion criteria were as follows: first metatarsophalangeal joint fusion, concomitant second or third tarsometatarsal joint fusion, double-level metatarsal osteotomy, proximal chevron metatarsal osteotomy, bunionectomy or Weil osteotomy only, history of fracture surgery on the foot during follow-up, and a follow-up period of less than 12 months. Following the inclusion and exclusion criteria, the remaining patients were subsequently divided into a modified DCMO group and a control (conventional DCMO) group.

A single, established senior foot and ankle surgeon performed all the cases. The two groups were conducted consecutively during respective periods. The patients in the control (conventional DCMO) group were treated from 2009 to 2015, while those in the modified DCMO group were treated between 2016 and 2021. The control group differed from the modified DCMO group in that the shape of the chevron cut was 60 degrees and was fixed with a 1.5 mm Kirschner wire (K-wire). Otherwise, the remaining procedures including lateral release, Akin osteotomy, and postoperative restrictions followed the same protocol in both groups.

### 2.2. Surgical Procedures

This modified technique is typically indicated for moderate hallux valgus deformities where the HVA is <40 degrees and first–second IMA is <15 degrees [15]. However, contraindications include instability at the forefoot or arthritis at the first metatarsophalangeal (MTP) joint [16].

Following spinal anesthesia, the patient was placed in a supine position. The pneumatic tourniquet was inflated just before the start of the operation. A lateral release with a separate incision proximal to the first webspace was performed before the bony procedures, including a transection of the intermetatarsal ligament and release of the adductor hallucis tendon and lateral suspensory ligament of the fibular sesamoid. Through this procedure, we attempted to remove what prevented the sesamoid bones from returning to their original position. Following the lateral release, the hallux was stressed in the varus direction to check for sufficient release.

**Figure 1:**
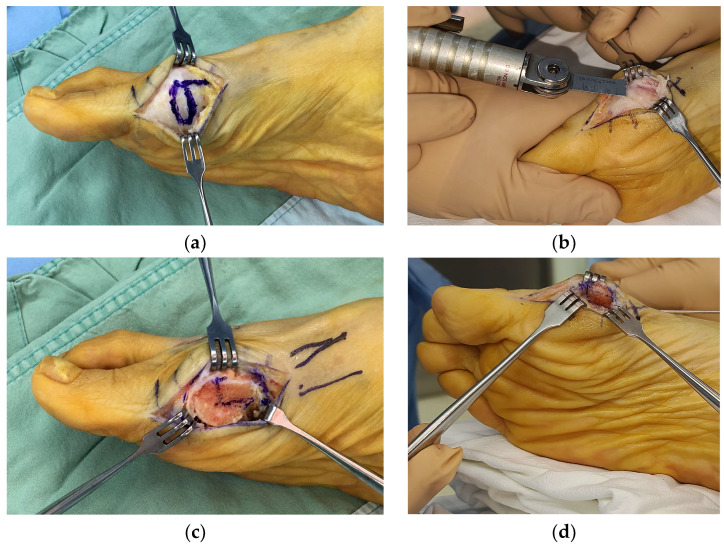

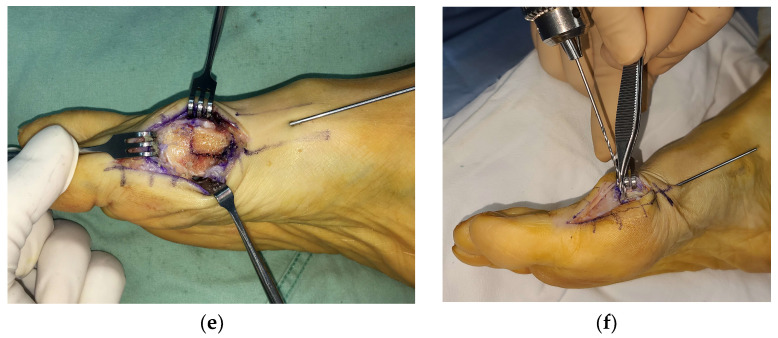
(**a**) Design of the incision of the capsule; (**b**) After the metatarsal head is exposed, resection of the medial eminence is performed; (**c**) Design of the distal chevron osteotomy angle; (**d**) Translation of the capital fragment; (**e**) Temporary fixation after osteotomy; (**f**) Fixation performed with a 2.4 mm cortical screw.

**Figure 2:**
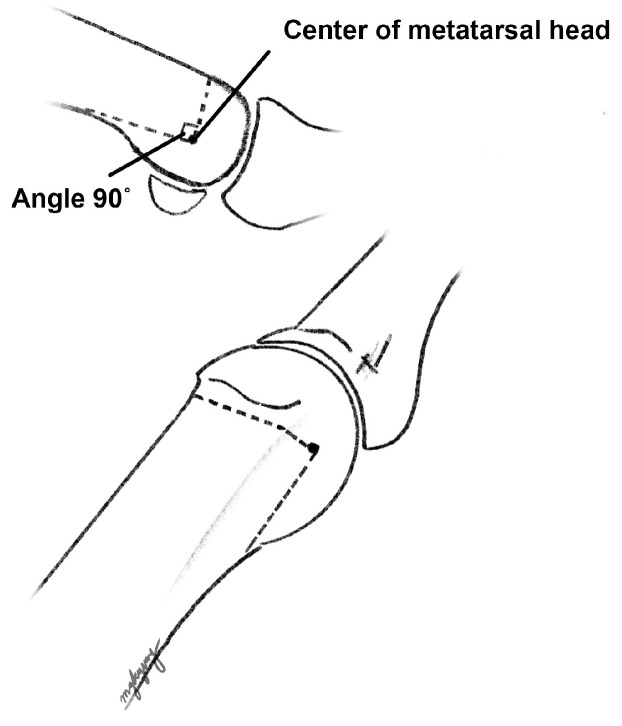
Schematic illustration of the modified technique. We utilized the osteotomy cut (dotted lines) with the angle at 90 degrees. Note that the exit of the transverse arm is located proximally to the capsule and provides a large contact area between the capital fragment and metaphyseal region of the metatarsal bone.

**Figure 3:**
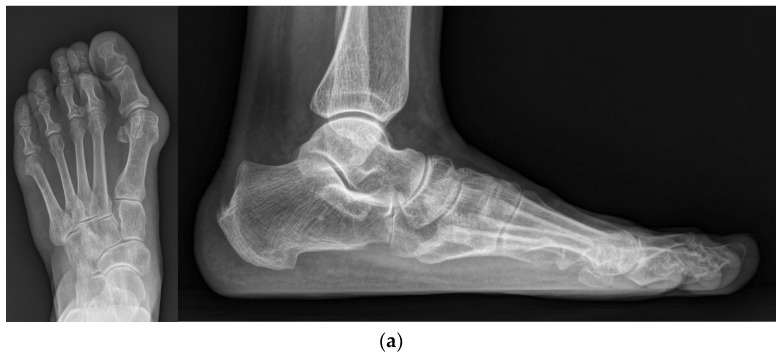

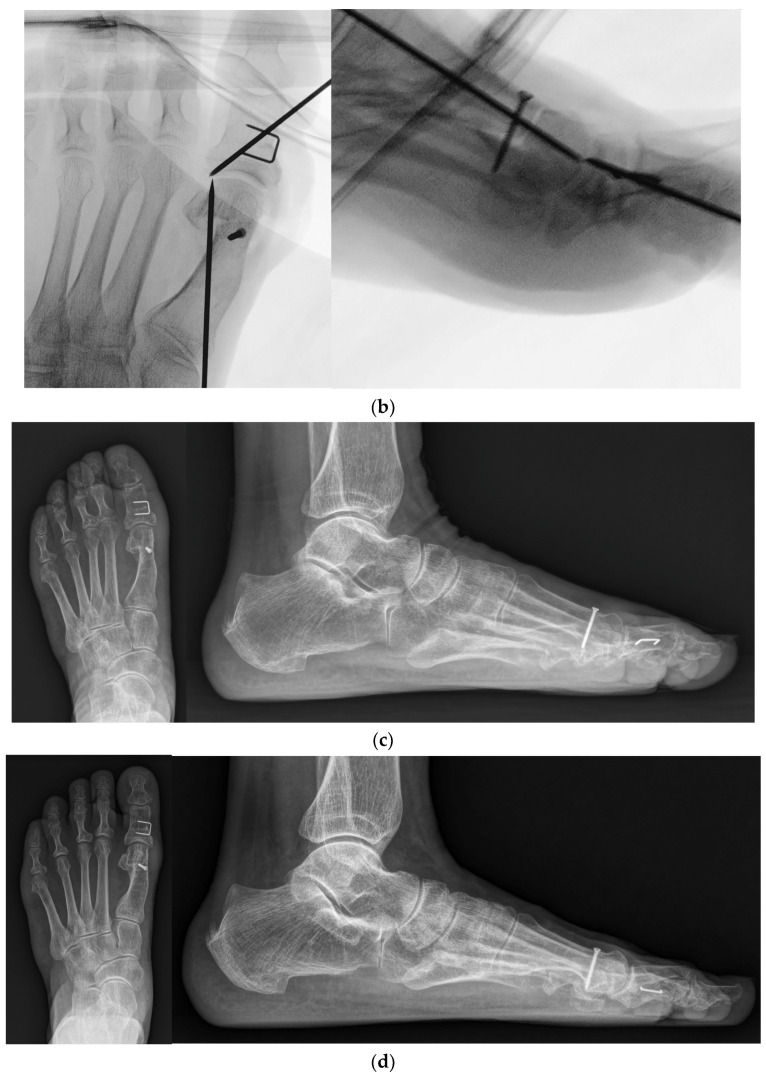
(**a**) Preoperative standing foot anteroposterior and lateral radiographs; (**b**) Intraoperative foot anteroposterior and lateral fluoroscopy images; (**c**) Standing foot anteroposterior and lateral radiographs at 2 months post operation; (**d**) Standing foot anteroposterior and lateral radiographs at the final follow-up.

A bunion at the medial side of the foot was palpated, and a medial skin incision was made along the metatarsal from the distal one-third of the metatarsal bone to approximately 1 cm distal to the MTP joint. A careful dissection was performed to avoid injuring the dorsal and plantar nerves. Once the capsule of the MTP joint was visualized, a hexagonal-shaped incision (Figure 1a) was created to expose the joint and medial protrusion. In addition, a 1 cm additional linear capsular incision was made along the metatarsal longitudinal axis extending from the apex of the hexagon. The thickened capsule was excised, and the protruded bunion was resected carefully using a saw (Figure 1b). After exposing the cancellous portion of the metatarsal head area, a 90-degree osteotomy plane was drawn (Figure 1c), followed by complete osteotomy with a saw. Using a small towel clip, the proximal metatarsal fragment was pulled medially, while the capital fragment was translated laterally to the desired degree of correction, and an attempt was made to maintain the impacted position between fragments (Figure 1d). Simultaneously, a temporary 1.5 mm K-wire was inserted from the proximal to distal direction to hold both fragments (Figure 1e). Then, a 2.4 mm cortical screw was inserted from the dorsal to plantar directions to achieve firm fixation (Figure 1f). A remnant bump on the medial side was trimmed using a saw. A schematic illustration of our modified technique is shown in Figure 2. A representative preoperative standing foot radiograph of the patient is shown in Figure 3a.

Following the modified 90-degree DCMO, a skin incision was extended distally to the level of the proximal phalanx diaphysis. A medial closing-wedge proximal phalangeal osteotomy, also known as Akin osteotomy, was performed using a saw. A temporary 1.5 mm K-wire was inserted from the distal to proximal directions. After pre-drilling the holes, a 10 mm stainless steel staple (QuickFix Staple System, Arthrex, Inc., Naples, FL, USA) was inserted for the fixation of phalangeal fragments. Intraoperative fluoroscopy was performed to ensure an appropriate length of the cortical screw, a proper location of the staple, and non-invasion of the MTP joint by the temporary K-wires (Figure 3b). In addition, Weil osteotomy was performed in other lesser toes, if needed.

After meticulous irrigation and hemostasis, medial plication and capsulorrhaphy were performed while the hallux was held in a proper position to additionally correct the remnant transverse and frontal plane deformities. A skin suture was performed, and protruded K-wires were cut and bent. Compressive dressing was performed by wrapping a rolled gauze to prevent the hallux from reverting to the valgus position, and a spacer was inserted between the first and second toes. A short-leg splint was applied in a neutral position.

### 2.3. Postoperative Management

Rehabilitation protocols were as follows: (1) At 2 weeks post operation, patients were advised to make an outpatient visit for the removal of the skin suture and temporary K-wires. Taping was applied to maintain the position of the hallux. Patients were allowed partial weight-bearing with the heel using crutches. (2) At 4 weeks post operation, patients were advised to remove the taping and splint. However, a postoperative shoe brace with a soft hallux valgus brace was worn for another month. (3) At 2 months post operation, the patients were instructed to sustain activities of daily living with a full weight-bearing and wear their own shoes (Figure 3c). Figure 3d shows the radiograph obtained at the final follow-up.

### 2.4. Radiographic Outcome Assessment

The HVA, first–second IMA, and DMAA were calculated from standing foot radiographs of the preoperative, postoperative 2 months, and final follow-up states by a single author using the picture archiving and communication system software (INFINITT PACS 7.0, INFINITT Healthcare Co., Seoul, Republic of Korea), as previously described [9,17]. The position of the medial sesamoid was also assessed using Hardy and Clapham’s method [18]. In addition, the first metatarsal head was checked for the presence of avascular necrosis following the operation. Lastly, recurrent hallux valgus was defined as an HVA >20 degrees, as previously described [19].

### 2.5. Statistical Analysis

Statistical analysis was performed using IBM SPSS Statistics for Windows, Version 25.0. (Armonk, NY, USA: IBM Corp.). The Kolmogorov–Smirnov test was used to determine the normal distribution of the data. Comparisons of the modified DCMO group and control group with regard to demographic parameters were based on a chi-squared test and Student’s *t*-test. With regard to radiographic measurement parameters, a paired *t*-test was used for pairwise comparisons between time frames (preoperative, postoperative 2 months, and final follow-up states), while Student’s *t*-test was used for comparisons between groups. In addition, a multiple regression analysis was performed to identify the influencing factors affecting the correction of the deformity. The primary outcome measure was the amount of correction of HVA, and the secondary outcome measure was the amount of correction of IMA. The patients’ ages and all measured radiographic parameters were set as continuous variables. The modified DCMO method was considered as “1” and the conventional method as “0”. The male sex was considered as “0”, whereas the female as “1”. Statistical significance was defined at the 5% (*p* < 0.05) level.

## 3. Results

**Figure 4:**
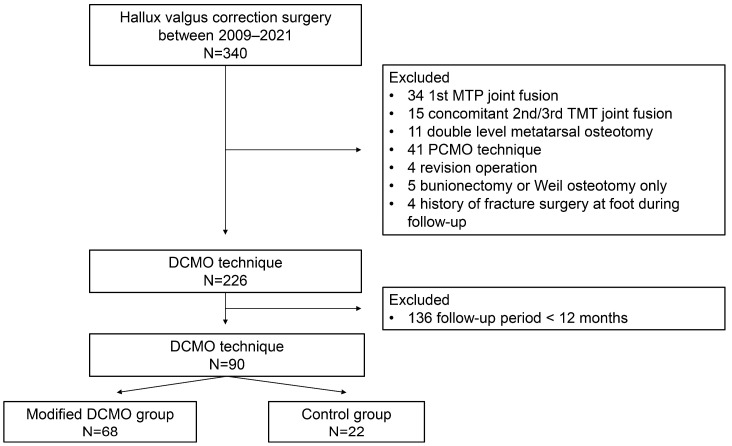
Flow diagram for the study patients. Abbreviations: DCMO, distal chevron metatarsal osteotomy; MTP, metatarsophalangeal; PCMO, proximal chevron metatarsal osteotomy; and TMT, tarsometatarsal.

A total of 90 feet were included in the analysis and subsequently divided into a modified DCMO group (n = 68 feet) and a control (conventional DCMO) group (n = 22 feet) (Figure 4). The patients’ demographic data are shown in Table 1. The age at the time of operation was the only difference between groups, which was significantly higher in the modified DCMO group (*p* = 0.002). The mean follow-up period for the modified DCMO group was 34.0 ± 15.4 (range, 13–65) months, while that of the control group was 35.7 ± 22.8 (range, 12–91) months (*p* = 0.688).

### 3.1. Radiographic Outcome

The comparison of preoperative radiographic parameters between the two groups demonstrated greater HVA in the modified DCMO group (*p* = 0.013) (Table 2).

When we compared the preoperative state and postoperative 2 months state, HVA, IMA, and DMAA were significantly decreased while the sesamoid position was significantly changed in both groups (Table 3). When the postoperative 2 months and final follow-up states were compared, there were also significant differences in HVA, DMAA, and sesamoid position in each group, but there were no significant changes in IMA in both groups (*p* = 0.887 and *p* = 0.089). Lastly, the final follow-up demonstrated significant correction compared to preoperative states in all of the radiographic parameters, where the amount of correction (HVA, IMA, and sesamoid position) was significantly greater in the modified DCMO group (Table 4).

In multiple regression analysis, age and preoperative HVA were found to be significant contributing factors to the amount of HVA correction. In addition, age, surgical method (modified DCMO technique), and preoperative IMA were found to be factors contributing to the amount of IMA correction (Table 5).

### 3.2. Postoperative Complications

Recurrence of hallux valgus occurred in 17 cases (25.0%) in the modified DCMO group and 5 cases (22.7%) in the control group at the final follow-up (*p* = 0.832). However, all 17 recurred cases in the modified DCMO group were significantly corrected (mean HVA of 24.6 ± 4.1 degrees, range 20.8–34.8 at the final follow-up) when compared to their severe preoperative deformities (preoperative mean HVA of 41.0 ± 7.0 degrees, range 29.1–51.8). On average, the HVA increased by 7.1 degrees from the postoperative 2 months state. None of the recurred cases underwent revision surgery in both groups.

No cases of AVN or nonunion appeared throughout the follow-up period in both groups. Furthermore, there were no cases of surgical site infection in both groups.

## 4. Discussion

DCMO is a popular procedure for the treatment of mild to moderate hallux valgus deformity [4]. Since the introduction of V-shaped chevron osteotomy (Austin procedure) in 1962 [20], various modifications have been reported, including the Youngswick, Kalish, and Johnson procedures [14,21,22]. Previous techniques have utilized the shape of chevron at 70 [14], 60 [22], or 55 degrees [21] for the osteotomy cut. Despite the fact that chevron osteotomy is a stable osteotomy and may not necessitate fixation, Jahss et al. reported that the loss of correction was observed in 12.5% of cases when there was no fixation to the osteotomy site [23]. In this way, mechanical stability between fragments can be problematic, possibly resulting in slower bone healing and loss of correction. However, in our modified technique, we utilized the osteotomy cut with the angle at 90 degrees and provided a large contact area between the capital fragment and the metaphyseal region of the metatarsal bone. Furthermore, the 2.4 mm cortical screw between the fragments was fixated perpendicular to the osteotomy plane. Together, these factors helped promote fast bone healing and enabled structural stability. This finding is also supported by a finite element analysis study by Matzaroglou et al., in which a 90-degree osteotomy showed a mechanical advantage over a typical 60-degree osteotomy [24]. They insisted that the compressive force that keeps the two fragments together is stronger while the shearing force that tends to slide between the two fragments is weaker in a 90-degree osteotomy.

In the modified DCMO group, the radiographic parameters (HVA, IMA, DMAA, and medial sesamoid position) were significantly corrected after the operation, and the corrected IMA was maintained until the final follow-up. Furthermore, the amount of correction was significantly greater when compared to the control group. In other words, our modified 90-degree DCMO technique enabled a large amount of correction, including DMAA and sesamoid positions. Specifically, based on the results of our multiple regression analysis, when preoperative HVA and IMA were greater, the corrections of HVA and IMA were also significant, respectively. They also demonstrated that the modified DCMO technique served as an important contributing factor to the correction of IMA. These results can be opposed to the idea that the correction angle of distal metatarsal osteotomy is limited compared to that of proximal metatarsal osteotomy [6]. Huang et al. reported that the majority of sesamoid corrections correlated with first–second IMA correction [10]. Moreover, some authors reported adding a biplanar component to DCMO to correct the DMAA [25]. In our modified technique, in addition to a lateral release with an attempt to remove what prevented the sesamoid bones from returning to their original position, the proximal metatarsal fragment was pulled medially in together, with the capital fragment displaced laterally and the impacted position between fragments maintained. We believe this resulted in not only the correction of the IMA and considerable change to the medial sesamoid position after the operation, but also in the correction of the DMAA by adding a biplanar component.

According to Hattrup and Johnson, up to 10% of the patients experienced recurrence after distal chevron osteotomy, which may be related to the under-correction of HVA [26]. However, the 17 recurrence cases in this study were moderate to severe deformities, with a preoperative HVA averaging 41.0 degrees. Although there was an average increase of 7.1 degrees compared to the postoperative 2 months case, all cases demonstrated a significant correction compared to their preoperative status. In a recent meta-analysis, it was reported that the prevalence of recurrence following a hallux valgus correction operation was 24.86% and the preoperative HVA showed a moderate positive relationship with recurrence [27]. Therefore, the 25.0% recurrence rate observed in this study can be considered comparable. Other than these cases, the 90-degree DCMO together with Akin osteotomy provided sufficient correction of the HVA and IMA, which led to favorable results.

The AVN of the metatarsal head occurs in 0–20% of cases following distal chevron osteotomy [11,12]. The resulting AVN is usually iatrogenic, which may be attributed to the disruption of intraosseous blood supply, excessive capsular release, and penetration of the lateral capsular vessels during sawing [11,12]. However, AVN did not occur in any of our case series during the follow-up. We believe that our technique does not have any unfavorable effects on the vascular networks near the osteotomy site because a sufficient bone stock is left in the capital fragment while the exit of the transverse arm is located proximally so that the capsule at the plantar side is minimally disrupted.

Interestingly, beyond the main finding of this study, patients in the modified DCMO group were older and had more severe preoperative HVA. This is in parallel with the context of population-based studies that have consistently shown a greater prevalence of hallux valgus in older individuals [28,29]. A possible reason for this could be that as age increases, the volume of the abductor hallucis muscle decreases [30], and there is a reduction in ligament stiffness and strength [31]. Nevertheless, just like other studies that reported no significant differences in postoperative outcomes for hallux valgus based on age [29], our study also showed reliable results in the modified DCMO group.

The modified technique presented herein provides several additional advantages. First, the procedure is simple and safe. A smaller amount of soft tissue stripping is required to perform corrective osteotomy than that required to perform proximal metatarsal or scarf osteotomy. In addition, a temporary K-wire removal can be easily performed in outpatient clinics. Furthermore, as we used a low-profile implant (2.4 mm cortical screw) for fixation, it does not necessitate implant removal and decreases the risk of irritation and surgical site infection. Second, this technique promotes fast bone healing and early weight-bearing, which consequently enable early rehabilitation and return to daily life with normal footwear.

Nowadays, minimally invasive chevron and Akin osteotomies (MICA) or percutaneous chevron and Akin osteotomies (PECA) for correcting hallux valgus have become increasingly popular. However, one of the most recent meta-analyses reported that MICA or PECA did not result in significantly improved clinical or radiographic outcomes when compared with conventional open surgery [32]. Additionally, because there is a learning curve for surgeons to become familiar with the new method, there has been concern about patients receiving the appropriate treatment during this period [32,33]. As an alternative, a previous study introduced the minimally invasive intramedullary nail device (MIIND) method, which allows for directly viewing the distal metatarsal through a mini-incision, reducing invasiveness while achieving a moderate amount or more of correction [34]. Their method has been shown to maintain its effectiveness in long-term follow-up periods of an average of 97 months. In our study’s modified DMCO group, we did not achieve the 23-degree HVA correction they demonstrated, but the HVA was significantly corrected compared to the preoperative status of our study group. The degrees of IMA and DMAA correction were comparable to theirs. However, they experienced superficial wound infections in nine patients (9%), and six (6%) of them eventually underwent removal surgery due to occasional pain or metal irritation. We did not encounter such issues, suggesting that our method has advantages compared to theirs. Furthermore, we believe that the detailed descriptions and figures of the surgical method presented in this study will greatly assist in reducing the learning curve.

On the other hand, there are some limitations to this study. First, as this study was performed retrospectively, there was a difference in the number of patients between the modified DCMO group and the control group, and there was also a discrepancy in the preoperative HVA degrees. However, within the numbers available, our data demonstrated favorable results. A prospective randomized clinical trial may be needed in the future study to compare outcomes between the two techniques and warrant the merits of our modified technique. Second, there would have been bias as a single author performed radiographic measurements. Lastly, this was a retrospective study, so we could not provide the functional outcome scores as there were many missing values. However, in order to evaluate the actual satisfaction of patients, these will need to be supplemented in future studies.

## 5. Conclusions

In this study, a modified 90-degree DCMO to correct moderate hallux valgus deformity was introduced, which demonstrated a comparable short-term radiographic outcome when compared to conventional DCMO. We believe our method is effective and safe, making it a reliable option among the choices for hallux valgus operations.

## Figures and Tables

**Table 1:** Patient demographic data.

	Modified DCMO Group (n = 68)	Control Group (n = 22)	*p* Value
Age, year	62.7 ± 8.4	55.2 ± 12.4	0.002
Sex, number	Male 2, Female 66	Male 2, Female 20	0.224
Side, number	Left 35, Right 33	Left 13, Right 9	0.533
Body mass index, kg/m^2^	24.5 ± 3.3	23.2 ± 2.5	0.099

Data are presented as mean ± standard deviation. Abbreviations: DCMO, distal chevron metatarsal osteotomy.

**Table 2:** Comparison of preoperative radiographic parameters between two groups.

	Modified DCMO Group (n = 68)	Control Group (n = 22)	*p* Value
Hallux valgus angle, degrees	36.1 ± 8.1	32.2 ± 5.5	0.013
Intermetatarsal angle, degrees	15.0 ± 3.8	14.2 ± 2.3	0.351
Distal metatarsal articular angle, degrees	21.6 ± 8.9	25.7 ± 7.6	0.058
Sesamoid position, grade	6.4 ± 0.6	6.1 ± 0.8	0.068

Data are presented as mean ± standard deviation. Abbreviations: DCMO, distal chevron metatarsal osteotomy.

**Table 3:** Radiographic outcome data.

	Preoperative	Postoperative 2 Months	Final Follow-Up	*p* Value *	*p* Value **	*p* Value ***
Modified DCMO group (N = 68)						
Hallux valgus angle, degrees	36.1 ± 8.1	11.8 ± 5.9	15.2 ± 7.3	<0.001	<0.001	<0.001
Intermetatarsal angle, degrees	15.0 ± 3.8	7.0 ± 2.6	7.0 ± 2.7	<0.001	0.887	<0.001
Distal metatarsal articular angle, degrees	21.6 ± 8.9	13.4 ± 5.3	14.8 ± 6.9	<0.001	0.022	<0.001
Sesamoid position, grade	6.4 ± 0.6	4.1 ± 1.0	4.4 ± 1.0	<0.001	<0.001	<0.001
Control group (N = 22)						
Hallux valgus angle, degrees	32.2 ± 5.5	14.2 ± 410	17.1 ± 5.1	<0.001	0.004	<0.001
Intermetatarsal angle, degrees	14.2 ± 2.3	7.6 ± 2.6	8.6 ± 2.9	<0.001	0.089	<0.001
Distal metatarsal articular angle, degrees	25.7 ± 7.6	12.4 ± 4.6	15.9 ± 4.4	<0.001	0.002	<0.001
Sesamoid position, grade	6.1 ± 0.8	4.5 ± 1.1	4.9 ± 1.1	<0.001	0.009	<0.001

Data are presented as mean ± standard deviation. * Results of paired *t*-test between preoperative and postoperative 2 months. ** Results of paired *t*-test between postoperative 2 months and final follow-up. *** Results of paired *t*-test between preoperative and final follow-up. Abbreviations: DCMO, distal chevron metatarsal osteotomy.

**Table 4:** Comparison of amount of correction between the modified DCMO group and the control group.

	Modified DCMO Group (n = 68)	Control Group (n = 22)	*p* Value
*Δ Hallux valgus angle, degrees	−20.9 ± 8.2	−15.1 ± 7.9	0.005
*Δ Intermetatarsal angle, degrees	−7.9 ± 3.2	−5.5 ± 2.7	0.002
*Δ Distal metatarsal articular angle, degrees	−6.8 ± 11.7	−9.8 ± 7.4	0.157
*Δ Sesamoid position, grade	−2.0 ± 0.9	−1.2 ± 1.1	0.002

*Δ refers to the difference between final follow-up and preoperative states. Data are presented as mean ± standard deviation. Abbreviations: DCMO, distal chevron metatarsal osteotomy.

**Table 5:** Effect of age, sex, surgical method, and radiographic parameters on the amount of hallux valgus angle and intermetatarsal angle correction by multiple regression analysis.

Dependent Variable	Independent Variable	Beta	95% CI	*p* Value
*Δ Hallux valgus angle	Constant	14.436	−2.950 to 31.822	0.102
	Age	−0.169	−0.312 to −0.027	0.021
	Sex	2.765	−3.978 to 9.508	0.417
	Surgical method	−2.249	−5.684 to 1.186	0.196
	Preoperative hallux valgus angle	−0.651	−0.846 to −0.457	<0.001
	Preoperative intermetatarsal angle	0.155	−0.275 to 0.584	0.476
	Preoperative distal metatarsal articular angle	−0.031	−0.193 to 0.131	0.702
	Preoperative sesamoid position	−0.513	−2.869 to 1.842	0.666
*Δ Intermetatarsal angle	Constant	3.977	−2.202 to 10.156	0.204
	Age	−0.069	−0.120 to −0.019	0.008
	Sex	1.285	−1.111 to 3.682	0.289
	Surgical method	−1.395	−2.616 to −0.174	0.026
	Preoperative hallux valgus angle	−0.002	−0.072 to 0.067	0.944
	Preoperative intermetatarsal angle	−0.553	−0.706 to −0.400	<0.001
	Preoperative distal metatarsal articular angle	0.032	−0.026 to 0.089	0.273
	Preoperative sesamoid position	0.041	−0.796 to 0.879	0.922

*Δ refers to the difference between final follow-up and preoperative states. Abbreviation: CI, confidence interval.

## Data Availability

The datasets generated during and/or analyzed during the current study are available from the corresponding author on reasonable request.
